# 

**Published:** 1892-01

**Authors:** 


### HALL'S JOURNAL of HEALTH

ESTABLISHED 1854

CONTENTS.

VOL. 39. No. 1.

| | PAGE. |
|---|---|
| The New Year | 1 |
| Scientific Killing | 1 |
| How it Seems to be Struck by Lightning | 2 |
| The Work of the Rain-Drop | 4 |
| Ants in Africa | 7 |
| An Affectionate Lion | 8 |
| A Strange Vision | 9 |
| A Story of Theodore Parker | 11 |
| Exercise for Elderly People | 11 |
| Slaughter of Girl Babies | 12 |
| Mazzini's Views of Death | 13 |
| Leanness | 14 |
| Destroying the Nerve in Decayed Teeth | 15 |
| Is the Sun a Magnet ? | 15 |
| The Hair | 17 |
| Sleep for Young People | 17 |
| Man's Seven Ages | 18 |
| Prayer Made Her Whole | 20 |
| Miscellaneous | 20 |
| Literary | 23 |
| Optimism | 24 |

INDEX TO ADVERTISEMENTS OF HALF A PAGE AND OVER.

| | PAGE. |
|---|---|
| A Christmas Gift | I |
| Hall's Journal of Health | II |
| Cornish & Co | IV |
| Erie Medical Co | V |
| Bovinine | VI |
| Dr. J. C. Ayer & Co | VII |
| Herba Vita | X |
| Woman's Work | XI |
| Hall's Journal Premiums | XII |
| Premiums Offered | XIII |
| Magee Emulsion Co | XIV |
| Rochester Lamp Co | XIV |
| Royal Baking Powder | XV |
| Buffalo Lithia Water | XV |

